# Mammographic Vascular Microcalcifications as a Surrogate Parameter for Coronary Heart Disease: Correlation to Cardiac Computer Tomography and Proposal of a Classification Score

**DOI:** 10.3390/diagnostics14242803

**Published:** 2024-12-13

**Authors:** Jonathan Andreas Saenger, Ela Uenal, Eugen Mann, Stephan Winnik, Urs Eriksson, Andreas Boss

**Affiliations:** 1 Diagnostic and Interventional Radiology, University Hospital Zurich, University Zurich, 8091 Zurich, Switzerland; 2 Institute of Diagnostic and Interventional Radiology, GZO Regional Health Center, 8620 Wetzikon, Switzerland; 3 Division of Cardiology, Department of Medicine, GZO Regional Health Center, 8620 Wetzikon, Switzerland; 4 Faculty of Medicine, University of Zurich, 8032 Zurich, Switzerland

**Keywords:** breast cancer, screening, mammography, coronary heart disease

## Abstract

Objective: This study develops a BI-RADS-like scoring system for vascular microcalcifications in mammographies, correlating breast arterial calcification (BAC) in a mammography with coronary artery calcification (CAC), and specifying differences between microcalcifications caused by BAC and microcalcifications potentially associated with malignant disease. Materials and Methods: This retrospective single-center cohort study evaluated 124 consecutive female patients (with a median age of 57 years). The presence of CAC was evaluated based on the Agatston score obtained from non-enhanced coronary computed tomography, and the calcifications detected in the mammography were graded on a four-point Likert scale, with the following criteria: (1) no visible or sporadically scattered microcalcifications, (2) suspicious microcalcification not distinguishable from breast arterial calcification, (3) minor breast artery calcifications, and (4) major breast artery calcifications. Inter-rater agreement was assessed in three readers using the Fleiss’ kappa, and the correlation between CAC and BAC was evaluated using the Spearman’s rank-order and by the calculation of sensitivity/specificity. Results: The reliability of the visual classification of BAC was high, with an overall Fleiss’ kappa for inter-rater agreement of 0.76 (ranging between 0.62 and 0.89 depending on the score). In 15.1% of patients, a BAC score of two was assigned indicating calcifications indistinguishable regarding vascular or malignant origin. In 17.7% of patients, minor or major breast artery calcifications were found (BAC 3–4). BAC was more prevalent among the patients with CAC (*p* < 0.001), and the severity of CAC increased with the BAC score; in the group with a BAC score of one, 15% of patients exhibited mild and severe CAC, in those with a BAC of two, this was 31%, in those with BAC of three, this was 38%, and in those with a BAC of four, this was 44%. The sensitivity for detecting CAC, based on the mammographic BAC score, was 30.3% at a specificity of 96.7%. Conclusions: The standardized visual grading of BAC in mammographies on a four-point scale is feasible with substantial interobserver agreement, potentially improving the treatment of patients with suspicious microcalcifications and CAC.

**Key Points:**

1. The classification of breast calcifications into (1) visible or sporadically scattered microcalcifications, (2) suspicious microcalcifications indistinguishable from breast arterial calcification, (3) minor breast arterial calcification, and (4) major breast arterial calcification is feasible with substantial inter-rater agreement and may improve patient management.
2. Breast arterial calcification on mammography correlates with coronary artery calcification (*p* < 0.001). Therefore, evaluation of potential coronary heart disease in patients with breast arterial calcification in mammography may be feasible and worthwile in large-scale mammography screening programs.

## 1. Introduction

Breast cancer and coronary heart disease (CHD) are among the leading causes of death in women worldwide [1,2,3]. Mammography screening programs have been shown to effectively reduce breast cancer mortality rates by detecting malignancies at a more curable stage, resulting in considerably reduced mortality rates from breast cancer [4]. The relative reduction of mortality from breast cancer by an organized screening program with regular screening examinations from 50 to 70 years is in the order of 25–40% [5]. In contrast to breast cancer, there are no systematic screening programs in place to reduce the mortality risk from CHD, which is globally responsible for an estimated 4.2 million deaths in women each year [6]. CHD clinically typically manifests in adults aged 65 and older, with more than four out of five cardiovascular disease-related deaths caused by heart attacks or strokes [7].

Breast cancer screening aims to detect a malignancy at an earlier stage compared to clinical findings to reduce mortality and to improve treatment options, allowing for a high number of patients undergoing breast-conserving therapy instead of mastectomy [8,9]. An proportion of 0–40% of breast cancers exhibit microcalcifications [10]. Microcalcifications (MC) are responsible for detecting 85–95% of cases of ductal carcinoma in situ (DCIS) on mammography screening [11,12]. MC are small calcium compounds with a size of 50–150 µm, typically calcium oxalate or calcium phosphate [13,14]. While calcium oxalate compounds are usually benign, calcium phosphate is associated with breast cancer [13]. The BI-RADS fifth edition lexicon considers ‘amorphous’, ‘coarse heterogeneous’, ‘fine pleomorphic’, and ‘fine linear or fine-linear branching’ as typically suspicious calcification morphologies [15]. Common secondary findings in mammograms are called Mönckeberg calcifications, which are breast arterial calcifications (BAC) involving the middle layer of the mammary arteries [16]. Previous studies have suggested that BAC seen on mammography may be correlated with coronary artery calcification (CAC), which is highly prevalent in patients with CHD [17,18,19,20].

The prevalence of calcifications in mammographies ranges from 9% to 17%, with increased prevalence with increased age [21]. Due to the high prevalence of MC in the age group mainly affected by breast cancer and CAC, the differentiation between BAC and suspicious microcalcifications is essential for further patient management. Although BACs are most often easy to identify, they can be challenging to differentiate from suspiciously grouped microcalcifications when only a few isolated calcified particles are visible at a single location, and their connection to a tubular structure is uncertain [22]. Therefore, these patients need to be identified, and additional imaging under compression with magnification may be required to clarify the nature of the microcalcifications further [22]. Furthermore, the classification of the MCs into BAC and potentially malignant MCs is essential to allow the identification of CAC-risk patients. Currently, there is no scoring system for BAC in the BI-RADS catalogue.

The aim of the present study is (i) to investigate the correlation between BAC calcifications in mammographies as a risk factor for the presence of CAC, (ii) to evaluate the radiological overlap of imaging findings in microcalcifications caused by breast cancer and precursors and microcalcifications from CAC, and (iii) to propose a suitable and stable scoring system from vascular microcalcifications analogous to the BI-RADS system.

## 2. Materials and Methods

### 2.1. Patient Selection

This retrospective analysis was approved on 6 July 2021 by the local ethics committee (No. 2021-01095), and all procedures were performed in accordance with local and federal regulations and the Declaration of Helsinki. All patients provided written consent prior to their inclusion in this study. This study included consecutive adult female patients who underwent cardiac computed tomography (CT) for suspected CAC between July 2012 and February 2024 at our institution. Patients included in this study were retrieved from our institutional picture archiving and communication system (PACS) and selected based on the availability of cardiac CT, including coronary artery calcium scoring and a routine screening mammography. Cases were defined as CAC cases if the disease had been confirmed by cardiac CT showing coronary artery calcifications with an Agatston score above 0.

### 2.2. CT-Imaging

Cardiac non-contrast CT scans were performed at our institution according to a standardized protocol. Additionally, the performance of contrast-enhanced coronary CT angiography was not reviewed for this study. Cardiac non-contrast scans were performed on a second-generation dual-source CT system (Siemens Somatom Definition Flash, Siemens Healthcare GmbH, Forchheim, Germany) between 2012 and 2013, on a third-generation dual-source CT system (Siemens Somatom Force, Siemens Healthcare GmbH, Forchheim, Germany) between 2014 and 2021, or on a first-generation dual-source photon-counting detector CT system (Siemens NAEOTOM Alpha, Siemens Healthcare GmbH, Forchheim, Germany) from 2022 onwards. Calcifications were assessed using the Agatston method and categorized into four subgroups based on the recommendations of Rumberger et al.: no calcification (Agatston score 0), minimal coronary artery calcification (Agatston score > 0–10), mild coronary artery calcification (Agatston score > 10–100), moderate coronary artery calcification (Agatston score > 100–400), and severe coronary artery calcification (Agatston score > 400) [23].

### 2.3. Mammography Protocol

Mammograms were performed at the University Hospital of Zurich or in a cooperating outpatient clinic according to the recommended mammography breast cancer screening protocols from the American College of Radiology. Two standard projections, craniocaudal and mediolateral oblique, were obtained for each breast [24]. Additional tomosynthesis, if performed, was not reviewed for this study. Mammography was performed using either the Senographe Essential Acquisition System ADS 56.21.3 (General Electrics, Boston, MA, USA), Image SDL or Class System (Internazionale Medico Scientifica/Giotto, Sasso Marconi, Italy), Selenia Dimensions (Hologic, Inc., Marlborough, NJ, USA) or by usage of a Mammomat Novation DR or Mammomat Inspiration System (Siemens Healthcare GmbH, Forchheim, Germany). In two cases, the mammography system could not be identified because of the retrospective digitalization of the images.

### 2.4. Definitions and Score Generation

**Figure 1:**
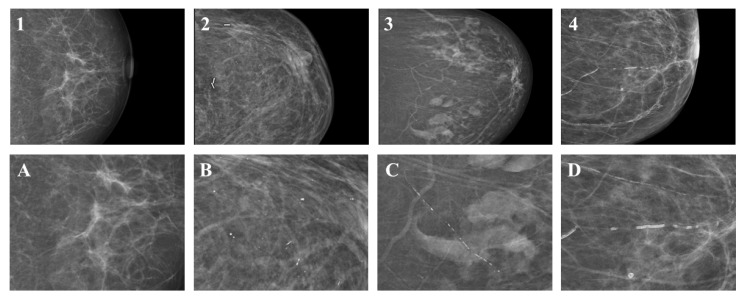
BAC Grade 1 consisted of patients without visible or sporadic scattered microcalcifications (Image **1**, magnification Image **A**). BAC Grade 2 was defined as grouped microcalcifications or less pronounced BAC mimicking grouped microcalcifications (Image **2**, magnification Image **B**). BAC Grade 3 was described as minor BAC (Image **3**, magnification Image **C**)., whereas BAC Grade 4 was defined as significant BAC (Image **4**, magnification Image **D**).

Microcalcifications were defined according to the fifth edition of the Breast Imaging Reporting and Data System (BI-RADS) [22]. As recommended, grouped microcalcifications were defined as at least five calcifications within 1 cm^3^ [22]. BAC, in general, was defined as linear tubular calcifications clearly associated with blood vessels [22]. Based on clinical analogy to the BI-RADS catalogue, four grades were established for microcalcifications and findings, possibly caused by breast artery calcifications (Figure 1): BAC Grade 1 consisted of patients without visible or sporadic scattered microcalcifications. BAC Grade 2 was defined as a few discontinuous calcified particles visible in a single location and with questionable association with a tubular structure mimicking fibroglandular microcalcifications. Calcifications clearly originating from BAC but of a modest degree were classified as BAC Grade 3, whereas BAC Grade 4 was defined as substantial BAC. All mammographies were read independently by three different readers (J.A.S., 3 years of experience; E.M., 10 years of experience; A.B., 20 years of experience). Each patient was assigned a grade if there were no visible microcalcifications, or if microcalcifications or BAC were detected in the right breast, the left breast, or both breast projections. Each reader gave the higher grade if the proposed grade differed between the right or left projections. For further evaluations in case of disagreement between readers, the majority of readers’ assigned scores were utilized. A consensus score was established through discussion if no majority was reached due to the divergence of the scores proposed by all readers.

### 2.5. Statistical Analysis

All analyses were performed using (SPSS, Version 29.0, Chicago, IL, USA). Continuous data were expressed as median with the range or interquartile range (IQR). Categorical data were expressed as numbers and percentages. We compared patient characteristics using the Pearson’s chi-square test or the Fisher–Freeman–Halton exact test with a two-tailed *p*-value of smaller than 0.05, considered statistically significant. To calculate the strength and direction of association between our proposed scoring system for BAC and the Agatston score as a quantitative parameter for coronary artery calcification, we used Spearman’s rank-order correlation. We used Cronbach’s alpha to evaluate the internal consistency of our proposed scale and calculated Fleiss’ kappa values for interobserver agreement to demonstrate feasibility and reproducibility. As proposed by Landis and Koch, kappa values of 0.20 or less indicated poor agreement; kappa values of 0.21–0.40, fair agreement; kappa values of 0.41–0.60, moderate agreement; kappa values of 0.61–0.80, substantial agreement; and kappa values of 0.81–1.00, great agreement [25]. Sensitivity and specificity were calculated using a 4-chamber analysis with two classes for BAC, taking together BAC grade 1 (no calcifications) and BAC grade 2 (unclear microcalcifications) as negative tests, whereas BAC grades 3 and 4 were taken as positive tests. An Agatston score evaluation resulting in no coronary artery calcification was regarded as a negative test, whereas minimal to severe coronary artery calcification was taken as a positive test.

## 3. Results

### 3.1. Patient Cohort

**Figure 2:**
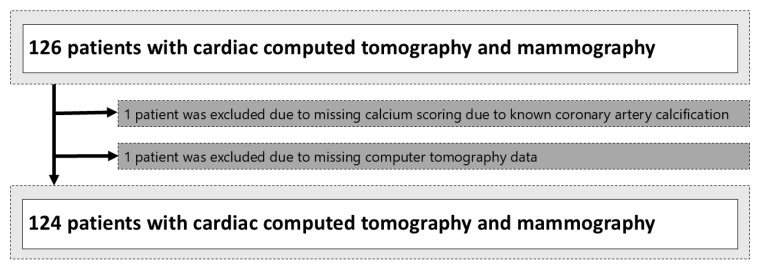
Flowchart illustrating inclusion and exclusion.

A total of 126 patients, having received both cardiac CT and mammography, which were available on our institutional PACS system, were included. Two patients had to be excluded, one because of missing calcium scoring due to known CAC and the other due to missing CT data (Figure 2). An overview of the patient characteristics is provided in Table 1. The median age was 57 years (IQR 15), with a significant difference between the VCs group with 62 years (IQR 12) and the no BAC group with 52 years (IQR 14). The median time difference between mammography and cardiac CT was 2.94 (IQR 17) years, with no significant difference between the two groups. Mammography of both breasts was available in 119 cases, whereas in 5 cases, due to ablation of the contralateral side, unilateral mammography was performed.

### 3.2. Cardiac CT Results

The majority of patients (53%) showed coronary artery calcification in cardiac CT, with a rate of 46% in the no-BAC group, which was significantly lower than the rate of 91% in the BAC group (*p* < 0.01). The Agatston score and the number of affected coronary vessels differed significantly between the two groups, as described in Table 2. Interestingly, of the affected coronary vessels, only the left anterior descending (LAD), right coronary artery (RCA) and circumflex artery (CX) were significantly more often affected in the BAC group compared to the no-BAC group, whereas no significant difference was observed for left main (LM) coronary artery.

### 3.3. Breast Densities and BI-RADS Classifications in Mammographies

Breast density distribution was (a) 15.3% (19 of 124); (b) 38.7% (48 of 124); (c) 33.9% (42 of 124); and (d) 12.1% (15 of 124), respectively. Most examinations were classified as BI-RADS 2 (105 of 124, 84.7%), followed by BI-RADS 3 (10 of 124, 8.1%) and BI-RADS 1 (5 of 124, 4.0%). Suspicious findings were described in 3.2% of the examinations (BI-RADS 4: 3 of 124, 2.4%; BI-RADS 5: 1 of 124, 0.1%).

### 3.4. Vascular Calcifications in Mammographies

When mammography detected vascular calcifications in breast tissue, they were bilateral in 18 cases, unilateral on the left side in 12 cases, and on the right side in 13 cases. After assessment by the participating radiologists, 83 patients were assigned a BAC Grade 1 for no visible or sporadically scattered microcalcifications. Nineteen patients were assigned BAC Grade 2 for clustered microcalcifications or milder BAC mimicking grouped microcalcifications. Thirteen patients were graded BAC Grade 3 because of minor BAC, while nine were graded BAC Grade 4 because of major BAC. Our proposed scoring system showed excellent internal consistency with a Cronbach’s alpha of 0.97. Overall, Fleiss’ kappa for inter-rater agreement showed a substantial agreement of 0.76. For BAC Grade 1, Fleiss’ kappa was the highest, demonstrating a great interobserver agreement at 0.89. BAC Grade 2 showed a substantial interrater agreement of 0.66. For BAC Grade 3, for minor BAC and BAC Grade 4 for major BAC, the interrater agreement was substantial at 0.62, respectively at 0.75.

### 3.5. Correlation Between Mammography and CT Findings

A significant association was seen between breast and coronary artery calcification (*p* < 0.001, Fisher–Freeman–Halton exact test). Spearman’s rank-order correlation showed a significant correlation between our proposed scoring system and the Agatston score as a surrogate parameter for coronary artery calcification (rs = 0.353, *p* < 0.001, n = 124) [26]. The sensitivity for predicting coronary artery calcification from the mammography BAC score was 30.3%, with a specificity of 96.7%, a positive likelihood ratio of 8.79, and a negative likelihood ratio of 0.72. The positive predictive value was 90.9%, and the negative predictive value was 54.9%. The number needed to screen in our population was approximately 2.66. This indicates that approximately three individuals must be screened using the mammography BAC score to identify one true case of coronary artery disease correctly.

## 4. Discussion

In our study, we delineated a scoring system to facilitate the differentiation of microcalcification and BAC and correlate the prevalence of BAC and CAC. We found that the classification of breast calcifications into (1) no visible or sporadically scattered microcalcifications, (2) suspicious microcalcifications indistinguishable from breast arterial calcification, (3) minor breast arterial calcification, and (4) major breast arterial calcification is feasible with excellent internal consistency and substantial inter-rater agreement. Moreover, we found a significant correlation between the presence of BAC in mammographies and CAC in cardiac CT.

**Figure 3:**
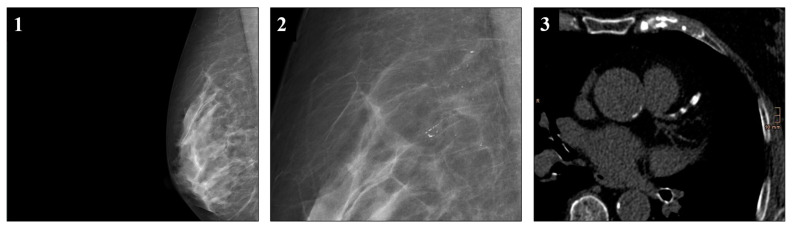
A 76-year-old female patient received screening mammography showing microcalcifications that were initially considered suspicious for malignancy in her left breast (Image **1**). Upon magnification (Image **2**), they appeared partly grouped and partly band-shaped. As a result of the microcalcifications being present in a previous mammogram conducted a decade earlier, it was deemed unnecessary to perform a further biopsy. A subsequent cardiac CT scan (Image **3**) conducted in the context of a planned transcatheter aortic valve (TAVI) implantation revealed considerable coronary artery calcification in the left anterior descending (LAD) and right coronary artery (RCA). This indicates that the microcalcifications observed in the mammogram may have been caused by breast arterial calcification, emphasizing the necessity for the comprehensive evaluation and further classification of breast arterial calcification.

The differentiation of BAC from malignant calcifications can be challenging as demonstrated in Figure 3. Prior case reports indicate that vascular calcifications were biopsied due to a lack of distinguishability [27,28]. Several attempts were made to grade the suspicious nature of microcalcifications to avoid unnecessary biopsy. In 2009, Maxwell et al. proposed a grading system for the further risk assessment of microcalcification by establishing the categories M1 (normal), M2 (benign), M3 (indeterminate/probably benign), M4 (suspicious of malignancy) and M5 (highly suspicious of malignancy) [29]. Currently, microcalcifications in mammograms are rated regarding their suspiciousness for the presence of breast cancer according to the American College of Radiology (ACR) Breast Imaging-Reporting and Data System (BI-RADS) system into categories ranging from one (no microcalcifications) to five (highly suspicious for breast cancer) [22]. So far, no current scoring system incorporates the classification of BAC in a grading system. Accordingly, we propose the presented scoring system to facilitate differentiation between BAC and suspicious microcalcifications and to visually grade the severity of BAC in mammographies analogues to other aspects of the BI-RADS catalogue. As previously described, patients without visible or sporadic scattered microcalcifications were assigned BAC Grade 1. BAC Grade 2 was defined as a few discontinuous calcified particles visible in a single location and with questionable association with a tubular structure mimicking fibro-glandular microcalcifications. Calcifications unquestionably derived from BAC, but of a relatively minor degree, were classified as BAC Grade 3, whereas BAC Grade 4 was defined as substantial BAC. As demonstrated, the visual evaluation of the classification system by a trained and experienced reader can be performed with substantial inter-rater agreement.

**Figure 4:**
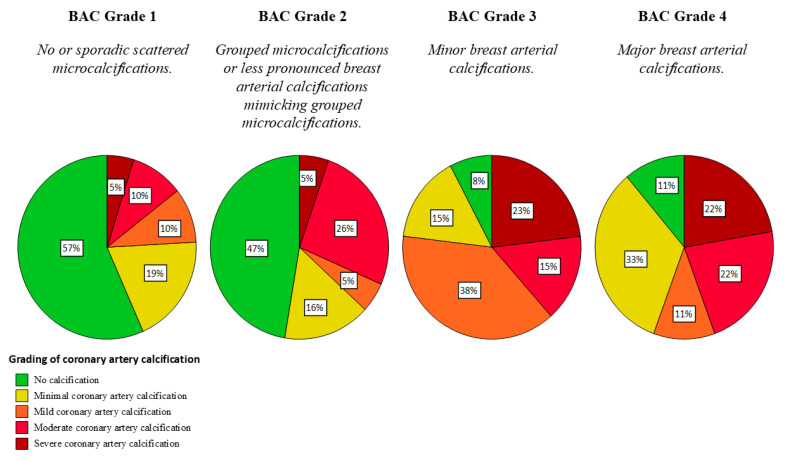
Distribution of the intensities of coronary artery calcification (CAC) depending on the grade of breast arterial calcification (BAC). Some percentages may not sum to 100% due to rounding.

Our analysis found that vascular calcifications in breast tissue seen via mammography correlated significantly with coronary artery calcification (Figure 4), as prior research had suggested. Maas et al. showed that 76% of the women with BAC had coronary artery calcification, which is lower compared to the 91% of the women we observed in our study [30]. The biological mechanisms underpinning the correlation between BAC and CAC include shared risk factors like age, diabetes, hypertension, and most likely hormonal changes, particularly post-menopause, which are known to influence both breast and arterial calcifications. Basically, inflammatory processes and disruptions in calcium metabolism that lead to calcification in breast tissue are similar to those seen in arterial walls. Common atherosclerotic mechanisms include lipid deposition, endothelial dysfunction, and plaque formation. Foam cell formation and the subsequent inflammatory cascade contribute to calcification in all arterial vascular beds. Whereas CAC predominantly affects the endothelial layer of the coronary arteries, BAC typically involves the middle layer of the mammary arteries, a common finding in general atherosclerosis of diabetics [16]. Accordingly, diabetes mellitus and hypertension are very strongly associated with BAC [31]. Notably, multiple studies have demonstrated that BAC itself is a predictor of CACs, independent of other known risk factors [17,19,32,33,34,35,36]. Therefore, identifying BAC in mammography might be superior to individual standard cardiovascular risk factors in identifying women with CAC [37].

With large mammography screening programs for breast cancer already in place in many countries, such as in the United States of America, with over 41 million mammograms annually, there is an additional huge potential for opportunistic screening for CVD in asymptomatic women without additional costs or risk [18,38]. Based on their results, Margolies et al. recommended further risk assessment of women with BAC by CT scan with calcium scoring, with subsequent risk factor adjustment, as indicated by the coronary artery calcium score [37]. Consequently, BAC screening may add to the selection of individuals with a high risk of cardiovascular events. Interventional studies aiming at primary CVD prophylaxis in this population are clearly warranted. There is potential to improve women’s health tremendously, since other opportunistic screening programs, for example, for coronary artery disease with low-dose computed tomography during lung cancer screening, might benefit men more than women [39].

Analogous to the BI-RADS system, we propose recommendations for potential follow-up management of patients based on the assigned grade. It can be reasonably presumed that no additional diagnostic procedures are required for patients with grade 1 with no BAC. In the case of BAC grade 2 with ambiguous presentation of microcalcifications, it may be advisable to consider a biopsy to rule out malignancy. Further cardiac work-up, if the biopsy turns out to be BAC, might not be necessary due to the limited and, therefore, confusable extent. If BAC grade 3 or 4 is present, it may be required to proceed with a detailed cardiovascular risk assessment, including further cardiologic work-up, where indicated.

The results must be interpreted in the context of the study design. First, the retrospective nature could have resulted in undetected confounding and selection biases. Second, the current study was conducted at a single institution, possibly reducing the robustness and generalizability of our data. Third, with our overall sample size of only 124 patients, the power of our study is limited, and the margin of error might be further reduced with a larger sample size. Fourth, the Agatson method was used to calculate coronary artery calcification, but age-adjusted percentiles were not calculated for the study population, resulting in a possible overestimation of coronary artery calcification. Fifth, the scoring of BAC in mammographies based on our proposed score was carried out in a retrospective study. Still, it may be assumed that a similar inter-rater agreement is also possible in a prospective evaluation of mammographies. Sixth, the interval between mammography and CT imaging averaged nearly three years, during which progression of atherosclerosis may have occurred, potentially influencing the observed correlations. Seventh, the absence of recorded major cardiac adverse events (MACE) during the follow-up period restricts the ability to evaluate the clinical implications of BAC and CAC as prognostic markers for coronary events. Eight, the absence of data from CT coronary angiography, invasive angiography, or functional tests to assess the functional significance of coronary artery calcification limits our ability to directly evaluate the ischemic relevance of CAC in our cohort, as increased CAC scores do not always correlate with clinically significant coronary artery disease causing myocardial ischemia. Future studies with larger cohorts, shorter imaging intervals, and the comprehensive tracking of MACE are needed to validate and expand upon our findings.

In conclusion, we showed in the presented study that breast arterial calcification in mammographies can reliably be classified according to a visual score analogues to other aspects of the BI-RADS classification system. The proposed BAC mammography score correlates with the coronary artery calcification measured with the Agatston score obtained from cardiac CT. As the new score can easily be applied in the evaluation of screening mammographies, it provides a tool for detecting female patients with coronary artery calcification, which could benefit from further diagnostic work-up or medical treatment to reduce the risk of myocardial infarction.

## Figures and Tables

**Table 1:** General and mammographic characteristics of 124 patients with and without breast arterial calcification (BAC).

Characteristic	Overall, N = 124	No BAC, N = 102	BAC, N = 22	*p*-Value ^1^
Age at mammography, median (IQR)	57 (15)	52 (14)	62 (12)	
Time between mammography and cardiac CT in years (IQR)	2.94 (17.0)	3.03 (15.8)	2.94 (17.0)	
Maximum diameter of breast vessels (mm)				
Left breast only	2.06	2.06	2.02	0.07
Right breast only	1.95	1.95	2.03	0.66
BAC laterality				<0.01
Unilateral	25 (20)	17 (17)	8 (36)	
Bilateral	18 (15)	4 (4)	14 (64)	
BAC Side				0.89 ^2^
Left breast only	12 (10)	8 (8)	4 (18)	
Right breast only	13 (10)	9 (9)	4 (18)	
ACR				0.25
a	19 (15)	13 (13)	6 (27)	
b	48 (39)	41 (40)	7 (32)	
c	42 (34)	34 (33)	8 (36)	
d	15 (12)	14 (14)	1 (5)	
BIRADs, n (%)				0.24
0	0 (0)	0 (0)	0 (0)	
1	5 (4)	5 (5)	0 (0)	
2	105 (85)	86 (84)	19 (86)	
3	10 (8)	9 (9)	1 (0)	
4	3 (2)	2 (2)	1 (5)	
5	1 (1)	0 (0)	1 (5)	

Median (IQR = Q3–Q1) or n (%) are shown unless otherwise specified. Some percentages may not sum to 100% due to rounding. BAC, breast arterial calcifications; ACR, American College of Radiology; BIRADS, Breast Imaging-Reporting and Data System. ^1^ Fisher–Freeman–Halton exact-test; ^2^ Pearson’s chi-square.

**Table 2:** Characteristics of coronary vessels of 124 patients with and without breast arterial calcifications (BAC).

Characteristics	Overall, N = 124	No BAC, N = 102	BAC, N = 22	*p*-Value ^1^
Maximum diameter of proximal coronary vessel (mm)				
LM	5.44	5.30	6.05	0.08
LAD	4.45	4.41	4.61	0.13
CX	4.25	4.16	4.64	0.68
RCA	4.57	4.50	4.89	0.02
Coronary Artery Calcification, n (%)	66 (53)	46 (45)	20 (91)	0.01
Number of affected coronary vessels, n (%)				<0.01
0	58 (47)	56 (55)	2 (9)	
1	29 (23)	22 (22)	7 (32)	
2	18 (15)	11 (11)	7 (32)	
3	12 (10)	8 (8)	4 (18)	
4	7 (6)	5 (5)	2 (9)	
Affected vessels				
LM	16 (13)	13 (13)	3 (13)	1
LAD	59 (48)	40 (39)	19 (86)	<0.01
CX	25 (20)	16 (16)	9 (41)	0.02
RCA	29 (23)	19 (19)	10 (41)	0.01
Agatston score, n (%)				<0.01
No CAC: CS = 0	58 (47)	56 (55)	2 (9)	
Minimal CAC: CS = 1–10	24 (19)	19 (19)	5 (22)	
Mild CAC: CS = 11–100	15 (12)	9 (9)	6 (27)	
Moderate CAC: CS = 100–400	17 (14)	13 (12)	4 (18)	
Severe CAC: CS > 400	10 (8)	5 (5)	5 (23)	

Median (IQR = Q3–Q1) or n (%) are shown unless otherwise specified. Some percentages may not sum to 100% due to rounding. BAC, breast arterial calcifications; CAC, coronary artery calcifications; IQR, interquartile range; LAD, left anterior descending; RCA, right coronary artery; CX, circumflex artery; LM, left main coronary artery ^1^ Fisher–Freeman–Halton exact test.

## Data Availability

The datasets presented in this article are not readily available since they are part of an ongoing study.

## Abbreviations

BAC	Breast arterial calcification
BI-RADS	Breast Imaging Reporting and Data System
CAC	Coronary artery calcification
CHD	Coronary heart disease
CT	Computed tomography
DCIS	Ductal carcinoma in situ
MC	Microcalcifications

## References

[1] BHF (2023). Global Heart & Circulatory Diseases Factsheet.

[2] Mehta L.S., Beckie T.M., DeVon H.A., Grines C.L., Krumholz H.M., Johnson M.N., Lindley K.J., Vaccarino V., Wang T.Y., Watson K.E. (2016). Acute Myocardial Infarction in Women. Circulation.

[3] Heron M. (2019). Deaths: Leading Causes for 2017. Natl. Vital Stat. Rep..

[4] Bleyer A., Welch H.G. (2012). Effect of Three Decades of Screening Mammography on Breast-Cancer Incidence. N. Engl. J. Med..

[5] Duffy S.W. (2006). Reduction in Breast Cancer Mortality from Organized Service Screening with Mammography: 1. Further Confirmation with Extended Data. Cancer Epidemiol. Biomarkers Prev..

[6] Sung H., Ferlay J., Siegel R.L., Laversanne M., Soerjomataram I., Jemal A., Bray F. (2021). Global Cancer Statistics 2020: GLOBOCAN Estimates of Incidence and Mortality Worldwide for 36 Cancers in 185 Countries. CA. Cancer J. Clin..

[7] Centers for Disease Control and Prevention (2012). Heart Disease Facts. Natl. Cent. Chronic Dis. Prev. Heal Promot. Div. Hear. Dis. Stroke Prev..

[8] Marcon M., Dedes K., Varga Z., Frauenfelder T., Boss A. (2018). Influence of breast cancer opportunistic screening on aesthetic surgical outcome: A single-center retrospective study in Switzerland. Breast J..

[9] Duffy S.W., Vulkan D., Cuckle H., Parmar D., Sheikh S., Smith R.A., Evans A., Blyuss O., Johns L., Ellis I.O. (2020). Effect of mammographic screening from age 40 years on breast cancer mortality (UK Age trial): Final results of a randomised, controlled trial. Lancet Oncol..

[10] Naseem M., Murray J., Hilton J.F., Karamchandani J., Muradali D., Faragalla H., Polenz C., Han D., Bell D.C., Brezden-Masley C. (2015). Mammographic microcalcifications and breast cancer tumorigenesis: A radiologic-pathologic analysis. BMC Cancer.

[11] Strax P., Venet L., Shapiro S., Gross S. (1967). Mammography and clinical examination in mass screening for cancer of the breast. Cancer.

[12] Nelson H.D., Fu R., Cantor A., Pappas M., Daeges M., Humphrey L. (2016). Effectiveness of Breast Cancer Screening: Systematic Review and Meta-analysis to Update the 2009 U.S. Preventive Services Task Force Recommendation. Ann. Intern. Med..

[13] Wilkinson L., Thomas V., Sharma N. (2017). Microcalcification on mammography: Approaches to interpretation and biopsy. Br. J. Radiol..

[14] Kenkel D., Varga Z., Heuer H., Dedes K.J., Berger N., Filli L., Boss A. (2017). A Micro CT Study in Patients with Breast Microcalcifications Using a Mathematical Algorithm to Assess 3D Structure. PLoS ONE.

[15] Spak D.A., Plaxco J.S., Santiago L., Dryden M.J., Dogan B.E. (2017). BI-RADS^®^ fifth edition: A summary of changes. Diagn. Interv. Imaging.

[16] Kim H., Greenberg J.S., Javitt M.C. (1999). Breast Calcifications due to Mönckeberg Medial Calcific Sclerosis. RadioGraphics.

[17] Oliveira E.L.C., Freitas-Junior R., Afiune-Neto A., Murta E.F.C., Ferro J.E., Melo A.F.B. (2009). Vascular Calcifications Seen on Mammography: An Independent Factor Indicating Coronary Artery Disease. Clinics.

[18] Zuin M., Rigatelli G., Scaranello F., Ribecco S.G., Picariello C., Zuliani G., Faggian G., Zonzin P., Roncon L. (2016). Breast arterial calcifications on mammography and coronary artery disease: A new screening tool for cardiovascular disease?. Int. J. Cardiol..

[19] Hendriks E.J.E., de Jong P.A., van der Graaf Y., Mali W.P.T.M., van der Schouw Y.T., Beulens J.W.J. (2015). Breast arterial calcifications: A systematic review and meta-analysis of their determinants and their association with cardiovascular events. Atherosclerosis.

[20] Liu W., Zhang Y., Yu C.M., Ji Q.W., Cai M., Zhao Y.X., Zhou Y.J. (2015). Current understanding of coronary artery calcification. J. Geriatr. Cardiol..

[21] Reddy J., Son H., Smith S.J., Paultre F., Mosca L. (2005). Prevalence of Breast Arterial Calcifications in an Ethnically Diverse Population of Women. Ann. Epidemiol..

[22] D’Orsi C.J., Sickler E.A., Mendelson E.B., Morris E.S., Creech W.E., Butler P.F., Wiegmann P.G., Chatfield M.B., Meyer L.W., Wilcox P.A. (2016). ACR BI-RADS^®^-Atlas der Mammadiagnostik.

[23] Rumberger J.A. (2008). Coronary Artery Calcium Scanning Using Computed Tomography: Clinical Recommendations for Cardiac Risk Assessment and Treatment. Semin. Ultrasound CT MRI.

[24] Houn F., Brown M.L. (1994). Current practice of screening mammography in the United States: Data from the National Survey of Mammography Facilities. Radiology.

[25] Landis J.R., Koch G.G. (1977). The measurement of observer agreement for categorical data. Biometrics.

[26] Cohen J. (1992). A power primer. Psychol. Bull..

[27] Janzen K., Janzen J. (2012). Arterial Microcalcifications in the Breast Mimicking Malignancy. Case Rep. Radiol..

[28] George J.T., Green L. (2021). Calciphylaxis of the breast, mimicking advanced breast cancer with skin involvement. Radiol. Case Rep..

[29] Maxwell A.J., Ridley N.T., Rubin G., Wallis M.G., Gilbert F.J., Michell M.J. (2009). The Royal College of Radiologists Breast Group breast imaging classification. Clin. Radiol..

[30] Maas A.H.E.M., van der Schouw Y.T., Atsma F., Beijerinck D., Deurenberg J.J.M., Mali W.P.T.M., van der Graaf Y. (2007). Breast arterial calcifications are correlated with subsequent development of coronary artery calcifications, but their aetiology is predominantly different. Eur. J. Radiol..

[31] Lee S.C., Phillips M., Bellinge J., Stone J., Wylie E., Schultz C. (2020). Is breast arterial calcification associated with coronary artery disease?—A systematic review and meta-analysis. PLoS ONE.

[32] Matsumura M.E., Maksimik C., Martinez M.W., Weiss M., Newcomb J., Harris K., Rossi M.A. (2013). Breast artery calcium noted on screening mammography is predictive of high risk coronary calcium in asymptomatic women: A case control study. Vasa.

[33] Iribarren C., Go A.S., Tolstykh I., Sidney S., Johnston S.C., Spring D.B. (2004). Breast Vascular Calcification and Risk of Coronary Heart Disease, Stroke, and Heart Failure. J. Women’s Health.

[34] Kemmeren J.M., Beijerinck D., van Noord P.A., Banga J.D., Deurenberg J.J., Pameijer F.A., van der Graaf Y. (1996). Breast arterial calcifications: Association with diabetes mellitus and cardiovascular mortality. Work in progress. Radiology.

[35] Schnatz P.F., Marakovits K.A., O’Sullivan D.M. (2011). The Association of Breast Arterial Calcification and Coronary Heart Disease. Obstet. Gynecol..

[36] Kemmeren J.M., van Noord P.A.H., Beijerinck D., Fracheboud J., Banga J.-D., van der Graaf Y. (1998). Arterial Calcification Found on Breast Cancer Screening Mammograms and Cardiovascular Mortality in Women: The DOM Project. Am. J. Epidemiol..

[37] Margolies L., Salvatore M., Hecht H.S., Kotkin S., Yip R., Baber U., Bishay V., Narula J., Yankelevitz D., Henschke C. (2016). Digital Mammography and Screening for Coronary Artery Disease. JACC Cardiovasc. Imaging.

[38] US Food and Drug Administration MQSA National Statistics. MQSA Insights 2022, 10–12. http://www.fda.gov/Radiation-EmittingProducts/MammographyQualityStandardsActandProgram/FacilityScorecard/ucm113858.htm.

[39] Gendarme S., Goussault H., Assié J.-B., Taleb C., Chouaïd C., Landre T. (2021). Impact on All-Cause and Cardiovascular Mortality Rates of Coronary Artery Calcifications Detected during Organized, Low-Dose, Computed-Tomography Screening for Lung Cancer: Systematic Literature Review and Meta-Analysis. Cancers.

